# CULTURALLY COMPETENT CARE FOR OLDER SEXUAL MINORITY ADULTS: A SYSTEMATIC REVIEW FOR HEALTHCARE DELIVERY

**DOI:** 10.1093/geroni/igz038.2718

**Published:** 2019-11-08

**Authors:** Debra Saliba, Sarah MacCarthy, Biayna Darabidian, Marc N Elliott

**Affiliations:** RAND Corporation, Santa Monica, California, United States

## Abstract

Recent attention to culturally competent care has largely overlooked the needs of older LGB adults. To address this, we conducted a systematic literature review and make recommendations for how the healthcare workforce can reduce sexual-orientation-based disparities. We searched PubMed, PsycINFO & CINAHL for manuscripts 1/1/10-6/19/18 (n=799), deduplicating, dually-screening abstracts (n=80), reviewing full-text articles (n=44), and classifying relevant articles (n=27) into five domains of cultural competency and associated recommendations: 1) Physical environment: display pictures with older same-sex couples and LGB-identified symbols; 2) Education/staffing: expand to include older-specific LGB issues, especially for key conditions (e.g., cancer, dementia,) and hire LGB-identified administrative/clinical staff; 3) Inclusive language and communication: review terminology on forms, electronic health records, and used with patients to ensure a broad range of terms (e.g., partner/spouse) and note older LGB may have more limited understanding/comfort with terminologies (e.g., self-identify as ‘something else’ instead of ‘gay/lesbian’ or ‘bisexual’); 4) Patient histories: discuss how factors particular to their sexual orientation (e.g., level of outness) may affect their support networks; 5) Subgroup differences: consider specific health concerns by sexual minority subgroups (e.g., healthy weight for lesbian women, HIV for gay men, and negative health outcomes for bisexual adults related to their simultaneous isolation from sexual minority and heterosexual communities) and note additional challenges based on characteristics such as race/ethnicity and urbanicity. Cutting across these domains are the ways in which local and national policies affect healthcare access and surrogacy (e.g., legality of same-sex partners to obtain health insurance, participation in medical decision making/visitation).

